# Sidewinder gait in horses

**DOI:** 10.1111/jvim.15870

**Published:** 2020-08-21

**Authors:** Monica Aleman, Emily Berryhill, Kevin Woolard, Charlotte A. Easton‐Jones, Tania Kozikowski‐Nicholas, Sue Dyson, Isabelle Kilcoyne

**Affiliations:** ^1^ Department of Medicine and Epidemiology University of California, Davis Davis California USA; ^2^ Department of Pathology, Microbiology and Immunology University of California, Davis Davis California USA; ^3^ The William R. Pritchard Veterinary Medical Teaching Hospital University of California, Davis Davis California USA; ^4^ Centre for Equine Studies, Animal Health Trust Newmarket United Kingdom; ^5^ The Cottage, Church Road, Market Weston Diss IP22 2NX United Kingdom; ^6^ Department of Surgical and Radiological Sciences University of California, Davis Davis California USA

**Keywords:** electrodiagnostics, equine, neurology, neurophysiology, species, spinal cord disease

## Abstract

**Background:**

Sidewinder gait in horses is poorly understood and characterized by walking with the trunk and pelvic limbs drifting to 1 side.

**Hypothesis/objectives:**

To report causes, clinical and diagnostic features.

**Animals:**

Horses examined at 2 institutions.

**Materials and Methods:**

Retrospective study (2000‐2019). Cases with sidewinder gait, neurological and orthopedic examination, and diagnostic work up or postmortem evaluation were included. Descriptive statistics were performed.

**Results:**

Twenty‐four horses (mean age 18.9 years) of various breeds and both sexes were included. Onset was acute (N = 10), subacute (N = 6), and insidious (N = 8). Electromyography and muscle biopsy supported neurologic disease and further aided in localizing site of lesion (N = 9/9). Neurologic causes included dynamic thoracolumbar spinal cord compression (N = 5), equine protozoal myeloencephalitis (N = 4, confirmed and presumed [2 each]), thoracic myelopathy of unknown etiology (N = 4), gliosis (N = 2), and thrombosis of thoracic spinal cord segments (N = 1). Non‐neurologic causes included osteoarthritis of the coxofemoral joint (N = 4), multiple displaced pelvic fractures (N = 2), bilateral rupture of the *ligamentum capitis ossis femoris* (N = 1), and severe myonecrosis of multiple pelvic limb muscles (N = 1). Case fatality was 79%.

**Conclusion and Clinical Importance:**

Sidewinder gait is usually observed in older horses and can have neurologic or musculoskeletal etiologies. Electromyography can be used as a diagnostic aid to determine neurologic versus non‐neurologic disease and further localize those of neurologic origin. The condition often has a poor prognosis for function and life.

**Abbreviations**
AAEP: American Association of Equine Practitioners
AHT: Animal Health Trust
AO: atlanto‐occipital
CSF: cerebrospinal fluid
EHV‐1: equine herpesvirus 1
EMG: electromyogram
EPM: equine protozoal myeloencephalitis
FP: fibrillation potentials
IFA: immunofluorescent antibody
LS: lumbosacral
PSW: positive sharp waves
TNCC: total nucleated cell count
VMTH: Veterinary Medical Teaching Hospital

## 1 INTRODUCTION

Sidewinder is a lay term used to describe horses with an unusual gait characterized by a disjointed movement of the thoracic and pelvic limbs, in which the trunk, pelvis, and pelvic limbs drift to 1 side while the thoracic limbs are usually normal.^1^ In severe cases, horses spin or circle in 1 place with their pelvic limbs moving in 1 direction while the thoracic limbs move in a compensatory manner. Other common names used for this gait include side walker and crab walker.^1^, ^2^ The complexity of this gait and apparent difficulty of affected horses to stand in 1 place, or to stand symmetrically loading the pelvic limbs, makes its investigation a diagnostic challenge. This syndrome is poorly understood and scarcely reported in the literature. In the opinion of the authors, this syndrome can have various etiologies that might range from musculoskeletal, neurological, to a combination of conditions resulting in this particular gait. A search of the peer reviewed English literature identified a single report of a 25‐year‐old Welsh Cob stallion with partial rupture of the *ligamentum capitis ossis femoris* as the cause of this gait.^2^ Anecdotally, this gait has been presumed to have a sudden onset in older horses, usually with a poor prognosis that often results in euthanasia. Furthermore, anecdotally some horses with more insidious onset problems have historically been awkward about picking up their feet for farrier work. Because of the limited information of this disorder, the purpose of this study was to describe the clinical and neurological findings, diagnostic results, and outcome of horses with sidewinder gait presented to 2 referral institutions and external consultation during the years of 2000 to 2019. The authors hypothesized that sidewinder gait has various etiologies including musculoskeletal, neurologic, or a combination of conditions resulting in this complex gait.

## 2 MATERIALS AND METHODS

The cases consisted of horses seen at the William R. Pritchard Veterinary Medical Teaching Hospital (VMTH) from the University of California at Davis, the Centre for Equine Studies, Animal Health Trust (AHT) at Newmarket, and external consultation from equine private practices from 2000 to 2019. The electronic medical records of the VMTH and the AHT were searched using the words sidewinder, side walker, crab walker, pelvic sway, drifting, leaning, pivoting, T3‐L3 myelopathy, T3‐caudal myelopathy, and lumbosacral (LS). The nomenclature of T3 or L3, for example, refers to spinal cord segments and caudal means spinal cord caudal to those spinal cord segments. Three main criteria had to be met to be included in the study: (1) horses presenting with a sidewinder gait; (2) having a full physical, orthopedic, and neurological examination; and (3) having a diagnostic work up or a postmortem evaluation. Sidewinder gait was defined as horses walking with their trunk, pelvis, and pelvic limbs drifting or leaning to 1 side while walking.^1^, ^2^ In severe cases, horses spin or circle with their pelvic limbs while their thoracic limbs move in a compensatory way.^2^ Horses with additional thoracic limb involvement were also included. Data retrieved from the medical record included signalment, history (onset, duration, progression), body posture (leaning, drifting, pivoting, dog sitting), posture of thoracic and pelvic limbs (limbs positioned in same or opposite direction), reluctance to move, stiffness, stilted gait, lameness and grade (American Association of Equine Practitioners [AAEP website, January 7, 2020^3^], see below), and apparent pain upon palpation. Onset of sidewinder gait was defined as acute (<48 hours), subacute (48 hours to 1 week), and insidious (>1 week). The neuroanatomical localization and severity of signs were recorded for horses classified as neurologically abnormal. Diagnostic data included hematology and biochemistry panel, and imaging modalities such as radiography, ultrasonography, nuclear scintigraphy, or computed tomography. Prospectively from 2012, additional diagnostic tests included intra‐articular anesthesia, electromyography (EMG), cerebrospinal fluid (CSF) analysis, and muscle biopsy. Not all diagnostics were performed in all cases. Treatment and outcome were also recorded. Short‐term survival was defined as survival to discharge. Of the horses discharged, follow‐up was completed by use of medical records or telephone conversation with the owner. Postmortem evaluation was recorded when available.

### 2.1 Lameness grading system

The grading system according to the AAEP guidelines^3^ consisted of 6 grades from 0 to 5 as follows. Grade 0 consisted of a lameness not perceptible under any circumstances; grade 1 difficult to observe and not consistently apparent regardless of circumstances; grade 2 difficult to observe at a walk or when trotting in a straight line but consistently apparent under certain circumstances; grade 3 lameness consistently observable at a trot under all circumstances; grade 4 lameness obvious at a walk; and grade 5 lameness with minimal weight bearing in motion, rest or both.

### 2.2 Neurological grading system

In brief, a modified grading system for signs of neurologic disease (eg, ataxia, proprioceptive dysfunction) as described by Lunn and Mayhew was used as follows.^4^ Grade 1: intermittent subtle abnormalities are visible during various tests including walking in tight circles, traversing different surfaces, and going up and down a hill or curb; grade 2: consistent mild deficits at all gaits and tests; grade 3: consistent moderate deficits easily visible by an untrained eye; grade 4: consistent severe deficits with risk of collapse and difficulty standing up; and grade 5: recumbency.^5^

### 2.3 Perineural and intra‐articular anesthesia

Perineural anesthesia of the tibial and fibular nerves, intra‐articular anesthesia of the 3 compartments of the stifle, and intra‐articular anesthesia of the coxofemoral joint were performed for all 4 horses seen at the AHT. A variable combination of nerve blocks was performed at the discretion of the attending clinician based on examination findings in the horses with suspected musculoskeletal disease seen at the VMTH.

### 2.4 Electromyography

Electromyography was performed using a Nicolet (Nicolet Viasys Healthcare Model, Viking Quest, Madison, Wisconsin). A concentric needle electrode (26G by 50 mm length, recording area of 0.07 mm^2^) was used to perform the EMG. A subdermal needle electrode (30G by 20 mm, Natus, F‐E2‐24 Genuine Grass Reusable Platinum Subdermal Needle Electrodes, 24″ wire) was used as a ground and placed close to the area of examination. The area of EMG examination was based on the neuroanatomical localization. Also, depending on ease of examination because of constant body leaning; other areas that were considered normal based on neurological examination were examined to confirm lack of abnormalities. When possible, the muscles examined bilaterally included the *splenius*, *trapezius*, *supraspinatus*, *infraspinatus*, *triceps brachii*, *longissimus* (*cervis*, *thoracicus*, and *lumbaris*), *multifidus* (*thoracicus* and *lumbaris*), *iliocostalis*, and *gluteus medius*. At least 5 sites per muscle were examined. Abnormal EMG was determined if decreased, prolonged, or absent insertional activity, and abnormal spontaneous activity such as fibrillation potentials (FP), positive sharp waves (PSW), complex repetitive discharges, and myotonic discharges were observed. The scoring system for EMG abnormalities was based on that used in human EMG as described by Kimura.^6^ In brief, 5 different areas of each muscle selected were examined and EMG findings were assigned grades 0 to 4 based on the following. Grade 0: no abnormalities in all areas examined; grade 1 (mild): mild numbers of FP or PSW in at least 2 of 5 areas examined; grade 2: moderate numbers of FP or PSW found in 3 areas; grade 3: many numbers of FP or PSW in all areas; and grade 4: severe abnormalities obscuring baseline in all areas.^6^ All EMG examinations and interpretation were performed by 1 of the authors (Monica Aleman). Sedation was not used to perform EMG except for 2 horses (xylazine hydrochloride at 0.25 mg/kg IV once) because of severity of signs and risk of collapse.

### 2.5 Histochemical evaluation of skeletal muscle

Muscle biopsies were collected for further investigation of possible cause(s) and definition of muscle atrophy as neurogenic versus disuse atrophy. Biopsies were collected from affected muscles according to EMG findings and other muscles at the discretion of the attending clinician. Lidocaine hydrochloride (1.5 mL) was applied SC as a local anesthetic. The method of collection included incisional or punch biopsy for superficial muscles and Bergstrom needle biopsy (AgnTho's AB, Lidingo, Sweden) for deeper muscles. Immediately after collection, the specimens were flash frozen in isopentane pre‐cooled in liquid nitrogen and stored at −80°C until further processing. The following stains and reactions were performed: hematoxylin and eosin, modified Gomori trichrome, periodic acid Schiff, phosphorylase, esterase, Streptococcal protein G‐horseradish peroxidase, ATPase at pH 9.8, 4.6, and 4.3, nicotinamide adenine dinucleotide, succinic dehydrogenase, acid phosphatase, alkaline phosphatase, and oil red O. Neurogenic muscle atrophy was defined by the presence of severe angular muscle atrophy of both fiber types (types 1 and 2). Disuse atrophy was identified based on the presence of type 2 myofiber anguloid atrophy.

### 2.6 Statistical analysis

Descriptive statistics were used in this study when applicable. Parametric data were presented as mean and SD, and nonparametric data as median and range.

## 3 RESULTS

Thirty‐seven horses with sidewinder gait were identified but 13 horses (suspected neurologic = 6, musculoskeletal = 7 horses) were excluded due to incomplete clinical information, no diagnostic workup, or lack of a postmortem examination. Twenty‐four horses were included in the study; 18 cases from the VMTH, 4 from the AHT, and 2 from external consulting (performed by Monica Aleman). Breeds included Quarter horse and related breeds (N = 13: Quarter horse N = 9, Appaloosa N = 3, Paint N = 1), Thoroughbred/Thoroughbred cross (N = 4), Warmblood (N = 1), Arabian (N = 1), Morgan (N = 1), Friesian (N = 1), Tennessee Walker (N = 1), Connemara (N = 1), and Highland pony (N = 1) breeds. These included 15 geldings and 9 mares. The mean age was 18.9 years old (SD ± 7.1 years). Ten horses had an acute onset of signs, 6 subacute, and 8 insidious. Progression of abnormal gait for those with insidious onset was reported to be slow. Time to presentation ranged from 2 to 180 days (median 14 days) after onset of sidewinder gait.

### 3.1 Physical examination and gait evaluation

**FIGURE 1:**
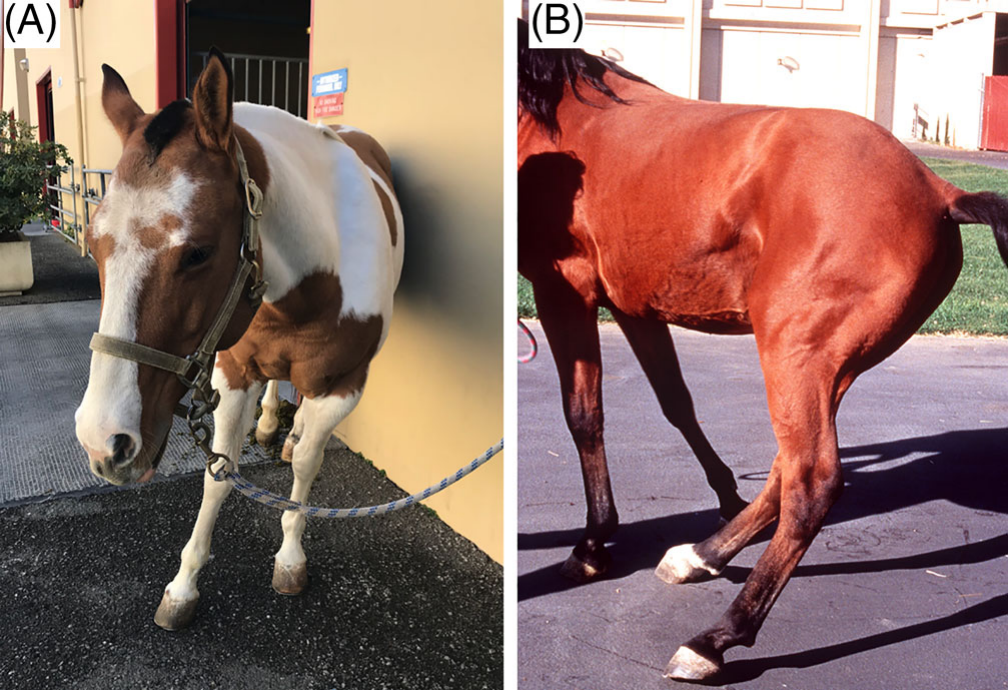
Clinical presentation: body posture. Note horses leaning excessively to 1 side while standing. A,B, Both horses were classified as having neurologic disease. B was published in Proceedings AAEP 2015^5^

Physiological variables were within reference range and no abnormalities other than the abnormal posture and gait were found upon physical examination. There was an apparent resistance or inability to pick up the pelvic limbs from the ground more profound in the limb supporting most of the weight in all horses. Fourteen horses appeared stiff and painful upon palpation of musculature and manipulation of the thoracolumbar (N = 7), pelvic (N = 4, bones), and coxofemoral joint (N = 3, 2 of which also had audible crepitus) areas. Fourteen horses had trouble laying down, and 7 had difficulty eating due to constant leaning and spinning to 1 side unless supported by a wall or fence (Figure 1).

### 3.2 Neurological and orthopedic evaluation

**FIGURE 2:**
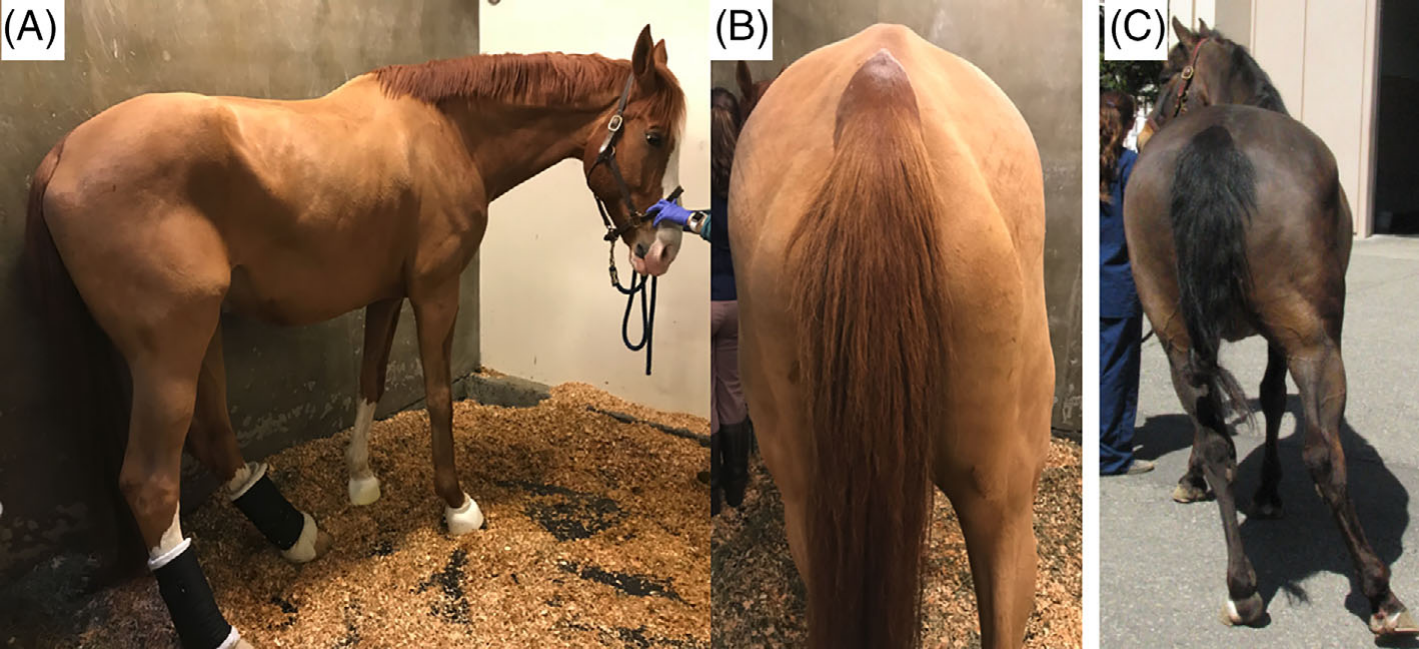
Clinical presentation: musculature. Note musculature of pelvic area and pelvic limbs in 2 horses with acute onset of sidewinder gait. Images taken weeks after onset: A,B = neurologic disease; C = musculoskeletal disease

Orthopedic and neurological evaluations were performed. The neurological examination in the majority of the horses (N = 17 of 20) was performed by 1 of the authors (Monica Aleman) as the primary or consulting clinician at the VMTH and by SD at the AHT. To further assess the posture and gait of these horses, archived videos for 19 horses were available for review. Based on examination, 16 horses were determined to have signs of neurologic disease, and the remaining 8 horses were suspected to have an underlying musculoskeletal problem. The mean age was 19.8 ± 7.9 and 17.1 ± 5.2 years for horses with neurologic and musculoskeletal disease, respectively. In horses with neurologic disease, the onset of abnormal gait was acute in 7, subacute in 6, and insidious in 3 horses. Horses with musculoskeletal disease had an acute (N = 3) and insidious (N = 5) onset. Fourteen horses were leaning to the left (neurologic N = 10, non‐neurologic N = 4) and 10 horses to the right (neurologic N = 6, non‐neurologic N = 4). Subjectively, decreased muscle tone and atrophy of the pelvic area and limbs appeared more profound in those horses classified as neurologic (Figure 2). Bilateral decreased muscle tone and atrophy were noted in 10 of 16 horses with neurologic disease which was more severe ipsilateral to the side of leaning. Six of 8 horses with musculoskeletal disease had unilateral muscle atrophy contralateral to the side of leaning.

The neuroanatomical localization of the horses classified as neurological (N = 16) was determined to be T3 to caudal myelopathy (N = 11), multifocal CNS disease (N = 4), and L4 to caudal myelopathy (N = 1). These horses had bilateral neurological deficits of the pelvic limbs but were drifting and walking sideways to the most affected side. Horses with multifocal disease also had brainstem (N = 2/4) and thoracic limb (N = 4/4) involvement. Three of 4 horses with multifocal CNS disease showed lower (weakness) and upper motor neuron signs in the thoracic and pelvic limbs, respectively. Weakness was seen as trembling and intermittent knuckling at the carpus and fetlock. Thoracic limb involvement was to a lesser grade (2 grades difference) than pelvic limbs according to a modified published grading system.^4^ Horses with pelvic limb ataxia were graded as 4 of 5 (N = 13) and 3 of 5 (N = 3). Pelvic limb placement for all 16 horses was consistently irregular.

Eight horses were determined to have primary musculoskeletal disease. Seven horses demonstrated a grade 4 of 5 lameness and were consistently walking side ways to 1 side. The 8 horses varied between grades 4 and 5 lameness. Abnormal placement of their pelvic limbs was consistently regular.

### 3.3 Clinical laboratory findings

Fifteen horses had a complete hematology and biochemistry panel, of which 14 horses had hematological and biochemical variables within reference values, and 1 had azotemia. Cerebrospinal fluid centesis was only performed in 11 horses because of safety concerns such as constant movement due to unsteadiness of the pelvic limbs and exacerbation of signs with sedation (atlanto‐occipital [AO] N = 8, C1‐C2 N = 1, LS N = 5 [3 horses had both AO and LS centesis). All AO CSF samples were collected immediately after horses were euthanized using an aseptic technique. Red blood cell count was low and ranged from 0 to 63 cells/μL. Total nucleated cell count (TNCC) was above reference range in 6 of 11 horses (TNCC from 4 to 5/μL, reference ≤3/μL). However, cell distribution was abnormal in 10 horses consisting of mild lymphocytic pleocytosis (N = 2, lymphocytes >75%), and mixed pleocytosis consisting of neutrophils, and small and large mononuclear cells (N = 8, neutrophils 7 to 44%). Protein concentration in CSF was above reference range in 3 of 10 horses (>80 mg/dL: 82, 83, and 111 mg/dL, respectively). Cerebrospinal fluid cytology from LS centesis was abnormal while cytology from the AO space was normal in all 3 horses which had both spaces evaluated.

### 3.4 Immunofluorescent antibody test

Immunofluorescent antibody (IFA) test for the detection of *Sarcocystis neurona* and *Neospora hughesi* antibodies was performed in 16 horses with signs of neurologic disease. Serum antibody titers for both protozoa were negative in 8 of 16 horses. One of these seronegative horses on both serum and CSF was later found to have *S neurona* upon histopathological evaluation. Four of 16 horses had IFA titers in serum for *S neurona* (80, 160, 640, and 1280) and negative titers in CSF; and 1 of these 4 horses also had IFA titers in serum for *N hughesi* (320) and negative titers in CSF. For 3 of these 4 horses, a definitive cause of sidewinder gait was identified and equine protozoal myeloencephalitis (EPM) was excluded. The remaining 4 horses had IFA titers in both serum and CSF presented as serum, CSF, and ratio as follows: horse 1 = *S neurona* 1280, 5, 256; horse 2 = 640, 5, 128; horse 3 = 80, 160, 0.5; and horse 4 = *N hughesi* 2560, 20, 128. A definitive cause was found in 2 of these 4 horses (N = 1 confirmed EPM based on histopathological evaluation and PCR, N = 1 gliosis at ventrolateral funiculus of T13 spinal cord segment and no histopathological evidence of EPM). The remaining 2 of 4 horses had presumed EPM as the clinical diagnosis despite a high serum to CSF ratio.

### 3.5 Intra‐articular anesthesia of the coxofemoral joint

After negative responses to distal limb perineural anesthetic blocks, intra‐articular anesthesia of the coxofemoral joint was performed in 4 horses seen at the AHT using 20 mL of mepivacaine. Synovial fluid was retrieved in all horses. Horses were assessed up to 1 hour after injection, but there was little alteration in gait. These horses were later diagnosed with severe coxofemoral arthritis (next sections).

### 3.6 Electromyography

**FIGURE 3:**
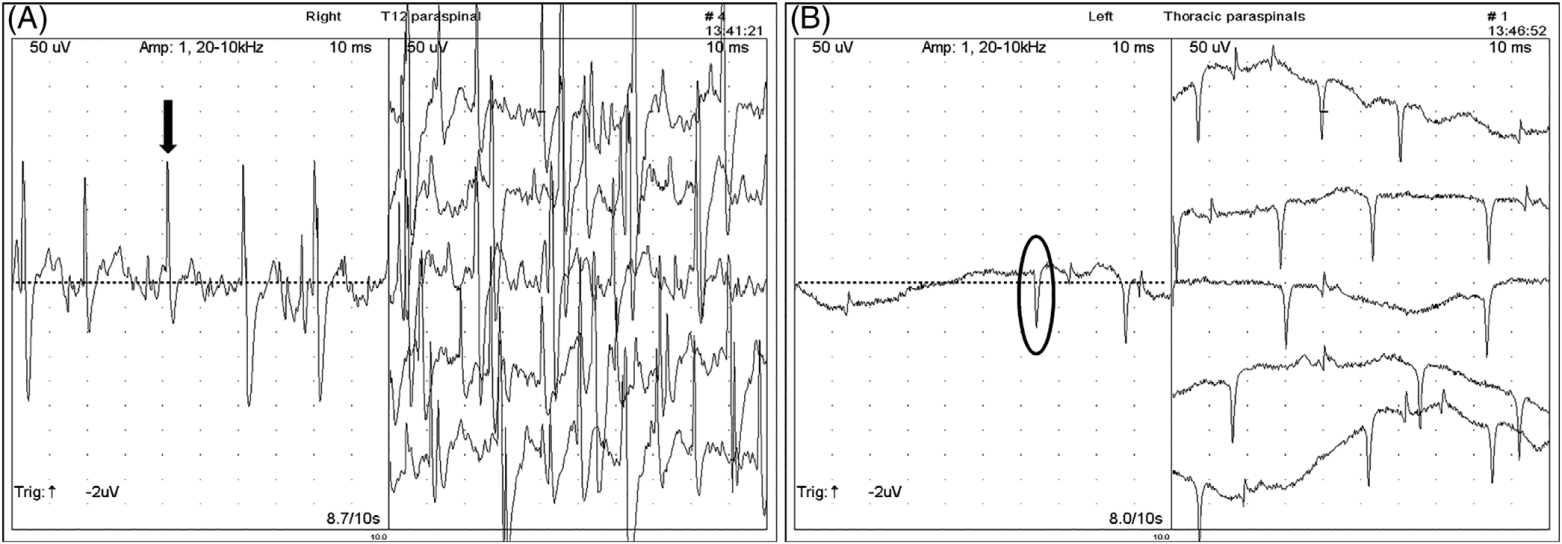
Electromyography. Note abnormalities of paraspinal muscles in 2 horses with neurologic disease of thoracic spinal cord segments. A, Series of fibrillation potentials (FP, single FP indicated by black arrow on left image). B, Series of positive sharp waves (PSW, single PSW indicated by black circle on right image). Note 50 μV and 10 ms per division

Electromyography was performed in 9 horses with signs of neurologic disease and abnormalities were found in all horses. Abnormal findings included prolonged insertional activity, FP (grade 3 to 4), and PSW (grade 3 to 4) (Figure 3). Abnormalities in paraspinal muscles were found bilaterally with 1 side more severely affected at the level of specific thoracic and lumbar spinal cord segments as follows within T16 to L3 (N = 3), T14 to T15 (N = 2), and T13 (N = 1). Three horses had multifocal asymmetrical thoracic, lumbar, and sacral spinal cord segments EMG abnormalities (2 horses had a clinical diagnosis of EPM but not confirmed).

### 3.7 Histochemical evaluation of skeletal muscle

After EMG examination, muscle biopsies were collected in 9 horses (1‐3 muscles per horse). The muscles samples collected included the *gluteus medius*, *biceps femoris*, *quadriceps*, *semimembranosus*, *longissimus lumbaris*, and *longissimus thoracicus*. The histological findings consisted of neurogenic muscle atrophy (N = 6 horses with neurologic disease), disuse atrophy (N = 2 with non‐neurologic disease), and severe myonecrosis (N = 1 non‐neurologic disease).

### 3.8 Imaging

Imaging consisted of radiography (N = 10), ultrasonography (N = 6), and nuclear scintigraphy (N = 5). Abnormalities observed on imaging included severe osteoarthrosis of multiple thoracic and lumbar articular process joints (N = 3 seen on radiography), lumbar and LS intervertebral disc disease (N = 2 identified using ultrasonography), severe osteoarthritis of the coxofemoral joint (N = 4 from the AHT), and multiple pelvic fractures (N = 2, ultrasonography). Two horses had moderately increased radiopharmaceutical uptake in the subchondral bone of the coxofemoral joint of the lame limb. There was unilateral increased radiopharmaceutical uptake in the region of the sacroiliac joint, tuber coxale, and sacrale in 1 horse. In 2 horses, radiopharmaceutical uptake pattern was normal. One horse with multiple pelvic fractures and involvement of the coxofemoral joint also had calcaneal retinaculum rupture resulting in lateral luxation of the superficial digital flexor tendon.

### 3.9 Therapy

Horses with signs of neurologic disease were treated with non‐steroidal anti‐inflammatory drugs (NSAIDs, N = 11), antioxidants (vitamin E N = 7, and C N = 4), antiprotozoals (ponazuril, N = 3), and intravenous fluids (N = 6). Posture improved slightly in 4 horses that received NSAIDs, 2 that received ponazuril but sidewinder gait remained. Four horses with musculoskeletal disease were treated with NSAIDs ranging from a few days to weeks. Initial mild improvement of the posture, apparent pain, and sidewinder gait was noted within a few days but severity of signs recurred while on treatment. Two horses with osteoarthritis of the coxofemoral joint received intra‐articular medication with methyl prednisolone acetate, but failed to respond.

### 3.10 Outcome

**FIGURE 4:**
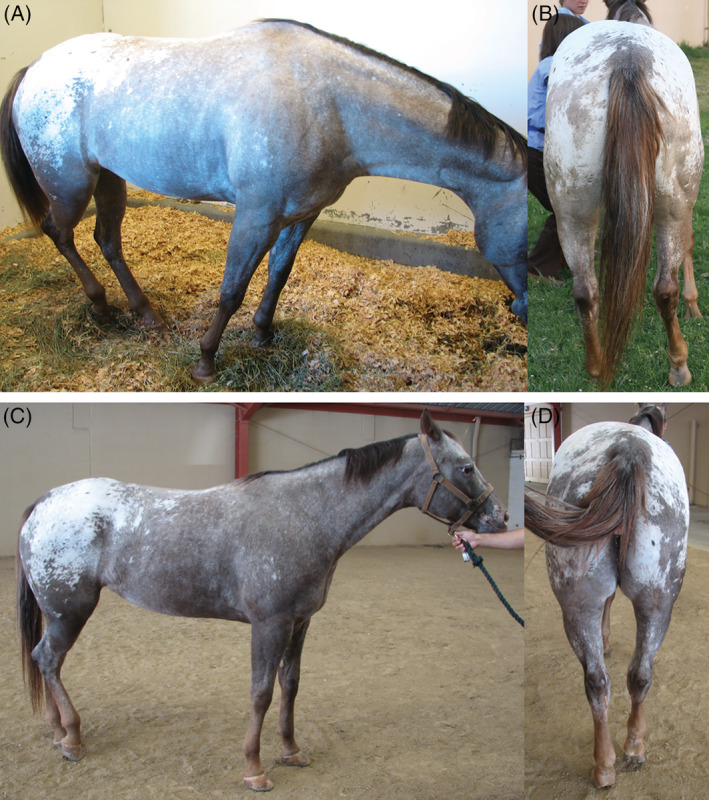
Clinical presentation and follow‐up. Appaloosa mare with presumed EPM on presentation (A,B; top images), and 90 days after treatment with ponazuril (C,D; bottom images). Note improved muscle mass and body posture but abnormal gait remained

Nineteen of 24 horses were euthanized (neurologic N = 12, non‐neurologic N = 7). Six horses were discharged alive (neurologic N = 5, non‐neurologic N = 1). Two horses with presumed EPM were discharged home on a 90‐day course of ponazuril (5 mg/kg orally once a day) and improved body posture, muscle mass, and 1 to 2 grades of ataxia and neurological deficits, but the sidewinder gait remained on follow‐up visits (Figure 4). One of these 2 horses was euthanized at home for a combined fatality rate of 79%. A follow‐up telephone call in the 5 remaining horses revealed no return to physical activity. Long‐term follow‐up (3 years) was available for 2 horses which were turned out in pasture with an improved but persistent sidewinder gait.

### 3.11 Postmortem evaluation

**FIGURE 5:**
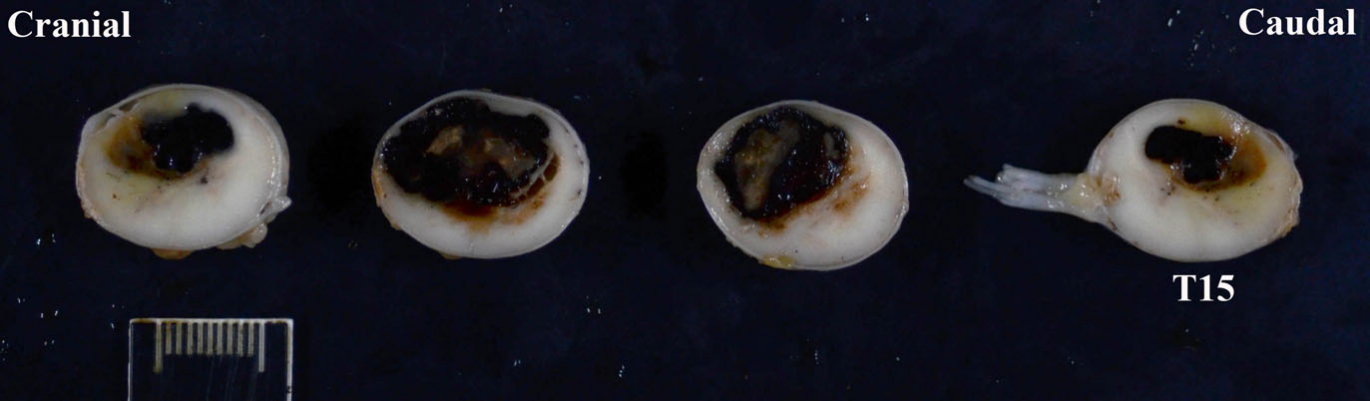
Postmortem evaluation. Note thrombus compromising up to 70% of the spinal cord segments from T12 to T15. Courtesy of Drs. Mohr and Rose at University of California, Davis

Postmortem evaluations were performed in 17 horses, of which 11 had neurologic and 6 musculoskeletal disease. In horses with neurologic disease findings included spinal cord compression based on histological evaluation and presumed to be from traumatic event or because of instability of the vertebral column (N = 5 [thoracic N = 2, lumbar N = 3 which also had chronic intervertebral disc disease]), histological identification of *S neurona* within the central nervous system (N = 2; 1 horse in thalamus and substantia nigra, 1 horse in thoracic spinal cord segments with malacia), ischemic injury due to thrombosis affecting a majority (70%) of the T14 to T15 spinal cord segments (N = 1, Figure 5), multifocal digestion chambers and gliosis in ventrolateral funiculi of unknown etiology at the level of T13 spinal cord segment (N = 1), and gliosis and lymphocytic perivascular cuffing of the brainstem and spinal cord suspected having a viral etiology (N = 1). Notably, the aforementioned horse with perivascular cuffing also had extensive fat saponification within the thoracic vertebral canal, which might have resulted in regional spinal cord compression. This horse also had atypical mineralized fat within the thoracic vertebral canal. The ventromedial and lateral tracts, and to a lesser extent the dorsal tracts were affected in horses with compressive myelopathy. The cause was not apparent in 1 horse with signs of neurologic disease, abnormal EMG and paraspinal muscle biopsy of the thoracic area.

Postmortem findings of non‐neurologic cases included advanced osteoarthritis of the coxofemoral joint of the lame limb but not the contralateral limb (N = 4), including 1 horse with partial rupture of the *ligamentum capitis ossis femoris*, bilateral *ligamentum capitis ossis femoris* rupture with roughening of articular surface of femur condyles and acetabulum and chronic osteoarthrosis more severe contralateral to the side of leaning (N = 1), and lymphocytic and histiocytic myonecrosis of unknown etiology affecting multiple muscles comprised mainly of pelvic limb abductors, contralateral to the side of side walking (N = 1).

### 3.12 Definitive diagnosis

Based on postmortem evaluation, a possible cause contributing to the gait was identified in 16 of 17 horses (neurologic N = 10, non‐neurologic N = 6). Based on a combination of diagnostic tests, a clinical diagnosis was made in the 8 remaining horses including the horse on which a postmortem evaluation did not identified a possible cause. Bilateral moderate to severe EMG abnormalities in muscles innervated by specific thoracic and lumbar spinal cord segments supported spinal cord disease in 9 horses. Two horses had EPM as the presumed clinical diagnosis based on progressive asymmetrical multifocal central nervous disease, seropositive IFA titers for *S neurona*, and evidence of neurogenic muscle atrophy of paraspinal muscles. Two with non‐neurologic disease had multiple displaced pelvic fractures with 1 case also involving the coxofemoral joint and 5 horses had advanced pathology of the coxofemoral joint.

## 4 DISCUSSION

Sidewinder is an uncommonly reported gait abnormality and, consistent with our testing hypothesis, might result from musculoskeletal and neurological causes. Although Quarter Horses and related breeds were the most represented breed in this study, this observation reflected the patient population at our VMTH institution. Older horses were more commonly affected as evidenced by a mean age of 19 years old. A sex predisposition was not identified in this small number of horses. Neurological diagnoses included compressive myelopathy at the thoracolumbar region presumed to be caused by trauma or vertebral instability; intervertebral disc disease; EPM; vascular disease; gliosis of the thoracic spinal cord segments of undetermined etiology; and lymphocytic perivascular cuffing suspected of viral origin. Horses with neurologic disease tended to walk drifting and leaning toward the most affected side with the contralateral limb abducted. These horses often placed the ipsilateral pelvic limb underneath the body and toward the thoracic limbs. This posture was exacerbated when horses supported their pelvis (ipsilateral to the side of leaning) on a wall or stocks. In severe cases, spinning of the pelvic limbs toward the side of the lesion and placement of the thoracic limbs underneath the body were commonly observed. Non‐neurological causes included osteoarthritis of the coxofemoral joint, multiple displaced pelvic fractures involving the coxofemoral joint, bilateral luxation of the coxofemoral joint due to rupture of the *ligamentum capitis ossis femoris*, and severe myonecrosis of multiple muscle groups (mainly abductors) of the pelvic limb. Horses with musculoskeletal disease walked drifting and leaning contralateral to the most affected side likely due to pain, mechanical inability to support the limb or both. Drifting away from the lame pelvic limb has previously been documented in association with a variety of sources of pain, but not as severely as observed in the current case series.^7^ There are no case series documenting coxofemoral joint osteoarthritis in adult horses, although there is a reference to the limb being rotated externally and carried abducted when advanced.^8^

Evaluation and identification of the cause of sidewinder gait is often a diagnostic challenge. The constant leaning and spinning to 1 side in some horses makes evaluation difficult. Sedation that might be needed to perform certain procedures can exacerbate leaning to the point of collapse, making further examination challenging or not possible. Furthermore, a clear distinction between neurological and orthopedic deficits could be difficult due to severity of gait abnormality and often unstable stance. In this study, EMG proved to be useful in the distinction of neurologic versus non‐neurologic disease and further aided in the localization of affected spinal cord segments. Cerebrospinal fluid centesis must be performed whenever possible closer to the lesion to increase the chances of finding abnormalities as shown in this study.^9^ Three horses had an AO CSF cytology and protein within reference range whereas those from LS CSF were abnormal. These 3 horses had vascular disease in thoracic spinal cord segments, thoracic compressive myelopathy, and presumed EPM with profound involvement of T3 to caudal spinal cord segments, each.

Different spinal cord diseases can affect a variety of specific tracts, sensory, or motor neurons, or all, depending of the site of injury. Sensory spinal cord deficits present as ataxia, abnormal proprioception and altered sensory input; whereas motor deficits manifest as paresis or paralysis, dysmetrias, and weakness.^10^ For instance, neuroaxonal dystrophy affects primarily sensory tracts and nuclei versus equine motor neuron disease affects lower motor neurons.^11^, ^12^ Compressive myelopathies regardless of cause (eg, cervical vertebral malformation, intervertebral disc disease, synovial cyst, mass‐occupying lesions such as neoplasia) affect both sensory and motor tracts. Both, sensory and motor deficits were noted in the horses of the current study. However, the motor tracts (ventromedial and lateral) were more profoundly affected causing paraparesis, bilateral dysmetria, severe weakness, and walking drifting and leaning ipsilateral to the most affected side in these horses.

Equine protozoal myeloencephalitis has been suspected to cause sidewinder gait but not reported nor confirmed.^1^ Here, we report 2 confirmed cases of EPM based on histopathological and molecular evaluation as the cause of the abnormal gait and posture. These 2 horses ages were 12 and 25 years of age. This disease more commonly affects horses younger than 4 years of age, whereas only 19.8% of cases are older than 8 years of age.^13^, ^14^, ^15^ Of interest, 1 of these 2 horses had negative IFA titers for *S neurona* and *N hughesi* on both, serum and CSF. This horse developed acute severe neurological deficits and presented to our hospital 4 days later. It is possible that an immune response was not fully developed at the time of testing due to its acute and severe course. This horse had severe necrotizing lymphocytic, histiocytic, and eosinophilic myelitis with malacia of several thoracic spinal cord segments. The second confirmed horse had a large number of *S neurona* schizonts and severe mononuclear perivascular inflammation in the thalamus and *substantia nigra* with no evidence of spinal cord involvement. The thalamus is a major relay motor center of the brain and the *substantia nigra* is an important part of the basal nuclei which control motor function.^10^ A possible explanation for this horse's abnormal gait could be the lack of proper upper motor input to the trunk, pelvis, and pelvic limbs more profound in the contralateral side to the leaning side. Another possibility is that histological lesions were missed in the thoracic spinal cord segments. Two additional cases were presumed to have EPM based on the presence of progressive asymmetrical multifocal spinal cord disease and positive IFA titers on both serum and CSF. However, IFA for *S neurona* serum to CSF ratios were high (128, 256; VMTH reference <64) in these 2 horses. These high ratios might indicate that intrathecal production of antibodies was not higher than that in serum as those described for surface antigen ELISAs (<100), making the clinical diagnosis of EPM less likely.^15^ Despite this possibility, both horses were treated with antiprotozoal medication based on their clinical presentation. Improvement of the posture and muscle mass was reported but the abnormal gait persisted in up to a year follow‐up. A definitive diagnosis was not reached in these horses.

The case with a thrombus involving T14 to T15 spinal cord segments was unusual in its presentation (ambulatory, grade 4 of 5) despite having 70% involvement of the spinal cord compromising sensory and motor tracts and nuclei at that location. This horse displayed both motor (paraparesis, dysmetria, weakness) and sensory (ataxia, abnormal proprioception, apparent focal hyperesthesia of thoracic region) deficits of the pelvic limbs. Vascular injuries of the spinal cord such as ischemic and hemorrhagic myelopathies and vasculitis have been described in horses.^16^, ^17^, ^18^, ^19^, ^20^, ^21^ Ischemic myelopathies are uncommonly reported in young horses usually resulting from fibrocartilaginous embolism at the C6 to T1 spinal cord segments and presumed to be from extruded nucleus pulposus which was not evident in these cases.^16^, ^17^, ^21^ Hemorrhagic myelopathies resulting in myelomalacia, inability to rise, and loss of pain perception are uncommon causes of severe neurological signs usually in young horses undergoing anesthesia for relatively short periods of time.^18^, ^19^, ^20^ Herpes myeloencephalopathy caused by equine herpesvirus 1 (EHV‐1) is a common cause of vasculitis, thrombosis, and hemorrhage of the spinal cord in horses.^22^ However, due to onset, long duration with slow progression of abnormal gait in the horses from this report, EHV‐1 infection was unlikely the cause of disease. Furthermore, horses with acute onset of neurological signs tested negative for EHV‐1 infection.

Electromyography was used in this study (9 of 24 horses) because it is a simple, easy, and minimally invasive diagnostic technique that aids in the detection of disease processes that affect the electrical activity of the muscle.^6^, ^23^ Although EMG does not provide a definitive diagnosis, its alterations can support a clinical diagnosis.^23^ The distribution of EMG abnormalities can assist in the localization of lesions to spinal cord segments, nerve plexuses, nerves, and muscles.^6^ EMG in this study helped in further localization of lesions within suspected spinal cord segments (eg, narrowing the clinical localization from T3 to caudal myelopathy to specific spinal cord segments) identified upon neurological examination in all horses tested. Multifocal abnormal spontaneous activity was identified in multiple spinal cord segments in a horse with EPM, 4 horses with thoracic and lumbar compressive myelopathy, T14 to T15 in a horse with vascular injury, and T13 in a horse with focal gliosis. Two additional horses had multifocal EMG abnormalities which cause remained unknown. EMG was not done in the remaining horses due to safety concerns.

Ultrasonographic examination was only possible in 6 horses due to the degree and exacerbation of leaning with sedation, and it was useful for the detection of multiple pelvic fractures in 2 horses with recent trauma, and intervertebral disc disease in 2 horses. Intervertebral disc disease could be an incidental finding since it has been observed in clinically normal horses.^24^, ^25^ However, in some cases it could be clinically relevant as a cause of nerve root pain, extradural compressive myelopathy, and more uncommonly fibrocartilaginous embolism, or both.^17^, ^24^, ^26^, ^27^

Characterization of pelvic fractures is essential for determination of prognosis.^28^ Ultrasonographic evaluation of the pelvis includes the coxofemoral joints, sacroiliac joints, and pubic symphysis and can be performed SC and transrectally in the standing horse.^28^, ^29^, ^30^, ^31^ Similar to the 1 case here, prognosis for multiple pelvic fractures particularly involving the acetabulum have the worst prognosis for returning to athletic performance.^28^ In 1 horse with multiple pelvic fractures from this report, severe sidewinder gait improved over a year but did not completely resolve. It is of interest that sidewinder gait was not reported in 2 studies of 75 and 86 horses with pelvic fractures.^28^, ^32^ The contribution of pelvic fractures to sidewinder gait in the horses from this report was not fully investigated since a postmortem evaluation was not performed.

The sidewinder gait was also observed in association with apparent musculoskeletal pain and exclusion of other potential causes as previously described.^2^ Bilateral degeneration of the articular cartilage of both the acetabulum and femoral head, and partial rupture of the left *ligamentum capitis ossis femoris* caused a sidewinder gait to the right in a 25‐year old Welsh Cob stallion.^2^ One horse from this report had bilateral coxofemoral luxation as the result of *ligamentum capitis ossis femoris* rupture with bilateral severe chronic osteoarthrosis causing leaning and side walking contralateral to the side with the more severe abnormalities; perhaps due to the inability to support weight on the most affected side as the result of complete luxation. However, a sidewinder gait was not reported in a study of 7 horses with coxofemoral subluxation presented with severe acute lameness (N = 6 grade 4 of 5, N = 1 grade 3 of 5) in which diagnosis was confirmed using ultrasonography.^33^ The diagnosis of coxofemoral subluxation required dynamic visualization of the femoral head being displaced from the acetabulum while placing weight on the affected limb and replacement into its normal position upon resting the limb.^33^ Acetabular rim fractures were also visualized in 6 of 7 horses; all had a poor outcome and 4 were euthanized.^33^ A postmortem evaluation performed in 2 of those horses showed elongation but intact *ligamentum capitis ossis femoris*.^33^

Furthermore, 5 horses in the current series had severe osteoarthritis of the coxofemoral joint which was confirmed radiographically in 4 and at postmortem evaluation in all horses. Radiographs were acquired with the horses standing as described (lateral dorsal 30° to lateral ventral oblique images) and also in dorsal recumbency under general anesthesia.^34^ The images acquired standing confirmed the presence of periarticular new bone, but much more detailed information about joint congruity, the extent of periarticular modeling and subchondral bone pathology was acquired from the images obtained under general anesthesia. The clinical relevance of the radiological abnormalities was supported by the presence of audible crepitus during passive manipulation of the coxofemoral joint in 2 horses and the results of ultrasonography in 2 other horses. A previous study demonstrated a similar ability to diagnose the presence of osteoarthritis of the coxofemoral joint using either radiography performed in standing horses or percutaneous ultrasonography.^35^ Intra‐articular anesthesia of the coxofemoral joint did not improve the gait in the horses from this report. Failure to improve is likely to be related to subchondral bone pain as observed in other joints.^36^

The cause of severe diffuse myonecrosis of muscles of the pelvis and pelvic limbs in 1 horse remained undetermined. Trendelenburg syndrome in humans results in hip abductor weakness and can have various etiologies.^37^ The *gluteus medius* and *profundus* are unilaterally weak and damaged resulting in the inability to maintain the pelvis in a normal position; therefore dropping the pelvis ipsilaterally and leaning when walking to the contralateral side.^37^ It is unclear here if something similar occurred to this horse.

Treatment in these horses was mainly supportive and palliative to address possible pain and inflammation, oxidative damage, and in some cases specific such as the use of antiprotozoal medication for those horses with presumed EPM. Although short‐term improvement was observed with therapy, the sidewinder gait remained unresolved. The fatality rate in this study was 79% (N = 19 of 24). Resuming previous physical activity was not achieved in the remaining live horses and long‐term follow‐up in 2 horses out in pasture revealed a persistent sidewinder gait.

In conclusion, sidewinder gait can have a variety of causes involving a neurologic, musculoskeletal, or both etiologies. Sidewinder gait is more commonly seen in older mature horses of any breed and sex. Sidewinders represent a diagnostic challenge due to excessive leaning, often constant spinning, instability while standing, and exacerbation of signs with sedation that might be needed to pursue diagnostics. The side of leaning might be ipsilateral or contralateral to the side of the injury depending on underlying cause, anatomical region, and severity. It appears from this small group of horses that prognosis for recovery of function is grave and poor for life. Electromyography proved to be useful to further localize lesions and aid in the determination of neurologic versus orthopedic causes. A major limitation of this study was the low number of cases with a definitive etiology. Furthermore, a cause and effect for the abnormal gait based on clinical and limited diagnostic findings in all the remaining horses was not proven.

## CONFLICTS OF INTEREST DECLARATION

Authors declare no conflict of interest.

## OFF‐LABEL ANTIMICROBIAL DECLARATION

Authors declare no off‐label use of antimicrobials.

## INSTITUTIONAL ANIMAL CARE AND USE COMMITTEE (IACUC) OR OTHER APPROVAL DECLARATION

An IACUC was not needed since the study was based on clinical cases with owners’ approval.

## HUMAN ETHICS APPROVAL DECLARATION

Authors declare human ethics approval was not needed for this study.
